# Molecular detection, phylogenetic analysis, and antibacterial performance of lactic acid bacteria isolated from traditional cheeses, North‐West Iran

**DOI:** 10.1002/fsn3.1887

**Published:** 2020-09-12

**Authors:** Mehran Hajigholizadeh, Karim Mardani, Mehran Moradi, Abdollah Jamshidi

**Affiliations:** ^1^ Department of Food Hygiene and Aquaculture Faculty of Veterinary Medicine Ferdowsi University of Mashhad Mashhad Iran; ^2^ Department of Food Hygiene and Quality Control Faculty of Veterinary Medicine Urmia University Urmia Iran

**Keywords:** functional food, phylogenetic analysis, probiotic bacteria, RFLP, traditional cheese

## Abstract

Lactic acid bacteria (LAB) are candidate probiotic bacteria that can provide health benefits when delivered via functional foods. The purpose of this study was to isolate and characterize LAB from traditional cheeses consumed in north‐west regions of Iran. A number of sixty traditional cheeses samples were collected and initially screened as LAB using biochemical and molecular methods. A fragment of 1,540 bp in size of 16s rRNA gene was amplified from 70 bacterial isolates. Restriction fragment length polymorphism (RFLP) was employed to differentiate LAB isolates. LAB isolates generated three different RFLP patterns using *Hinf*I restriction enzyme. Phylogenetic analysis revealed that LAB isolates belonged to three genera including *Enterococcus*, *Lactobacillus*, and *Lactococcus*. Most of the isolated LAB strains belonged to *Enterococcus* spp. The antimicrobial performance of eight LAB isolates with different RFLP patterns ranged from 6.72 to 14.00 mm. It was concluded that molecular characterization of LAB strains in traditional cheeses will enhance our understanding of traditional food microbiota and will help to find bacterial strains with probiotic potential with great benefit both in health and industry.

## Graphical abstract

Most of the isolated LAB strains belonged to Enterococcus spp. The antimicrobial performance of some LAB isolates with different RFLP pattern ranged from 6.72 to 14.00 mm

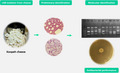

## 1 INTRODUCTION

The present evidences emphasize the health benefits of some microorganisms. In this regard, probiotics are currently among the most studied beneficial microorganisms (Cassani, Gomez‐Zavaglia, & Simal‐Gandara, 2020). Lactic acid bacteria (LAB) are a diverse group of microorganisms, having the ability to produce lactic acid as the main product, which prevents the proliferation of food spoilage bacteria and pathogens. They are Gram‐positive, nonspore forming, cocci or rods, with the ability to secrete a variety of antimicrobial compounds, for example, organic acids, bacteriocins, etc. (Quigley et al., 2013). LAB are normal inhabitants of the healthy gut microbiota in animals and can be found in the milk and dairy products such as cheese.

Lactic acid bacteria are the most commonly used probiotics in the food. Nowadays, the development of novel probiotic‐based foods has gained more attention. Probiotics display several health benefits by affecting the intestinal flora and permeability, modulating the immunological parameters in the body, and producing bioactive or regulatory metabolites (George et al., 2018). Nowadays, there is a global interest to isolate LAB from food commodities for application in functional food and dietary supplement (Chiang & Pan, 2012). LAB isolated from different food sources drawn a lot of attention in combating food‐associated pathogens and spoilers, biodegradation of chemical contaminants, and development of unique food with special interests for the consumers (Koohestani, Moradi, Tajik, & Badali, 2018; Lim, Yeu, Hong, & Kang, 2018). LAB secrete diverse bioactive metabolites such as organic acids, short‐chain fatty acids, carbohydrates, antimicrobial peptides, enzymes, vitamins, cofactors, immune‐signaling compounds, and complex agents, and most of them, for example, organic acids, hydrogen peroxide, bacteriocins, etc. exhibit different antibacterial activity in individual and synergistic forms on foodborne pathogens (Moradi et al., 2020). The bactericidal effects of such agents on a wide range of pathogens such as *Listeria monocytogenes*, *Escherichia coli,* and *Staphylococcus aureus* have been studied (De Vuyst & Leroy, 2007; Hassanzadazar, Ehsani, & Mardani, 2014).

To date, various food sources, for example, human and animal milk, cheese, meat products, etc. have been investigated for isolation and identification of new LAB strains with novel functional properties (Al‐Gamal et al., 2019; Awaisheh & Ibrahim, 2009; Hassanzadazar et al., 2014). This has resulted in the acquisition of new strains. However, researchers are still trying to isolate and identify new isolates with unique properties from different sources. Koopeh, Shal, and Shoor are three popular traditional cheese varieties consumed in the North‐West of Iran. Koopeh cheese is one of the most popular fermented dairy food not only in Iran but also in some parts of Turkey and Iraq (Amirbozorgi, Samadlouie, & Shahidi, 2016). It is a semisoft type cheese made mainly from raw ewe's milk and less commonly from cow milk or a mixture of both kinds of milk without using any starter in clay jugs (Hassanzadazar et al., 2014). Shoor is a salty cheese that traditionally is produced from boiled buttermilk with a reasonable amount of salt, and Shal cheese is another popular milk product in the west of Iran which is freshly prepared in large casts.

In the present study, we attempted to isolate and discriminate LAB from three traditional cheese varieties consumed in West Azerbaijan province, Iran, using biochemical and molecular methods. The phylogenetic tree of the isolated LABs was generated based on the partial sequences of the 23s rRNA gene. Additionally, the antibacterial performance of LAB isolates was investigated on *L. monocytogenes*.

## 2 MATERIAL AND METHODS

### 2.1 Cheese collection

Sixty traditional cheese samples (~200 g) composed of Koopeh (*n* = 20), Shal (*n* = 20), and Shoor (*n* = 20) were collected from markets from three different geographical locations in West Azerbaijan province, Iran. The samples were aseptically transferred to the Faculty of Veterinary Medicine, Urmia University, under cold and aseptic conditions.

### 2.2 Bacteria isolation and biochemical characterization

First, an amount of 25 g of each cheese sample was added to 225 ml 0.1% w/v peptone water (Merck, Darmstadt, Germany) and homogenized using a stomacher (Seward Medical Ltd., London, U.K) at 280 rpm for three min. The cheese suspension was diluted in 2% w/v sodium citrate (Merck) and cultured on two De Man, Rogosa and Sharpe (MRS) agar (Quelab, Montréal, Canada) plates and incubated under anaerobic (anaerobic jar) and aerobic conditions for 1–2 days at 37°C. The 3–4 different single colonies were randomly selected from each cultured plate. The selected colonies were Gram stained, examined microscopically, and catalase test was also performed. Gram‐positive and catalase‐negative bacilli/cocci were chosen and stored in cryotube containing 15% (v/v) glycerol (Merck) at −20°C for further molecular and antibacterial characterization.

### 2.3 Molecular characterization

#### 2.3.1 Extraction of bacterial genomic DNA

Genomic DNA from previously cultivated LAB in MRS broth (Quelab) for 12–18 hr at 37°C was used. The DNA was extracted using DNA extraction kit (SinaClon, Tehran, Iran) according to the kit's manufacturer instruction. The quality and amount of extracted DNA were evaluated by Nano‐Drop 2000c (Termo Scientific, Massachusetts, USA). Then, extracted DNA samples were kept at −20°C for subsequent application.

#### 2.3.2 Amplification of 23s rDNA

A pair of primers EGE1 (5′‐AGAGTTTGATCCTGGCTCAG‐3′) and EGE2 (5′‐CTACGGCTACCTTGTTACGA‐3′) described by Sharifpour, Mardani, and Ownagh (2016) was used for the amplification of a fragment on 1,540 bp in size. Polymerase chain reaction (PCR) was performed in 25 µl reaction volume, containing 0.5 µl Taq DNA polymerase (5 U per µl), 1 µl MgCl_2_ (50 mM), 0.5 µl of each primer, 4 µl of dNTP (1.25 mM), 2.5 µl of 10X PCR buffer, and 4 µl (50–100 ng) of genomic DNA. Thermal conditions for PCR were as follow: Initial denaturation at 94°C for 5 min, followed by 32 cycles of denaturation at 94°C for 45 s, annealing at 55°C for 45 s, elongation at 72°C for 1.25 min, and a 5 min final elongation at 72°C. PCR products were electrophoresed in 1.5% w/v agarose gel and visualized by Ultraviolet (UV) transilluminator (Synoptics, Cambridge, UK) after staining with Safe Stain (Sharifpour et al., 2016).

#### 2.3.3 Purification and restriction endonuclease digestion of PCR products

All amplified PCR products were subjected to purification using Combo GP Kit (GeneALL, Seoul, Korea) following the kit's manufacturer instruction. For restriction endonuclease digestion of purified PCR products, *Hinf*I endonuclease (Jena Biosciense, Germany) was used. Digestion reaction was performed in 15 µl reaction volume, containing 1 µl FastDigest *Hinf*I enzyme, 1.5 µl universal buffer, 4.5 µl dH_2_O, and 8 µl of the purified PCR product. The reaction mixture was incubated at 37°C for 5 min. The digested PCR products were electrophoresed, stained with Safe Stain, and visualized using a UV transilluminator.

#### 2.3.4 DNA sequencing and phylogenetic analysis

Purified PCR products of eight bacterial isolates with different restriction fragment length polymorphism (RFLP) patterns were selected for further nucleotide sequencing. Purified PCR products from the gel were sequenced (Bioneer Company, Daejeon, Korea). The obtained nucleotide sequences of 16S rRNA were BLAST searched in GenBank (National Centre for Biotechnology Information, Rockville Pike, Bethesda, USA) using the advanced BLAST similarity search option and compared to the 16S rRNA sequences of *Lactococcus*, *Lactobacillus,* and *Enterococcus* subsp. from GenBank (Table 1). Nucleotide sequences were aligned and compared to other nucleotide sequences retrieved from GenBank using Clustal W, and the phylogenetic tree was generated using the neighbor‐joining method in MEGA software (version 6.0; Biodesign Institute, Tempe, USA) (Kumar, Stecher, Li, Knyaz, & Tamura, 2018).

**TABLE 1:** 16s rRNA sequences of lactic acid bacteria retrieved from GenBank

	LAB	Accession Number		LAB	Accession number
1	*Ent. durans* strain KLDS 6.0930	KF768355.1	11	*Lb. farciminis* strain KJ05	KX139185.1
2	*Ent. faecium* strain G141	EF204317.1	12	*Lb. farciminis* JCM 1,097	LC063168.1
3	*Ent. durans* strain XT1−1	MH891655.1	13	*Lb. farciminis* strain KCKC 3,681	CP017702.1
4	*Ent. faecium* strain VKU17	MK640929.1	14	*Lb. delbrueckii* subsp. *Bulgaricus* strain LGM2	AY675257.1
5	*Ent. faecalis* strain PUFSTSe	MN396189.1	15	*Lb. acidophillus* strain IDCC 3,301	EF533992.1
6	*Lac. lactis* strain NWAFU3004	MG551180.1	16	*Lb. casei* strain RKG 1−188B	MT045986.1
7	*Lac. lactis* strain RKG 2–790	MT045240.1	17	*Lb. paracasei* strain SMVDUDB5	MN865202.1
8	*Lb. plantarum* strain IMAU80162	GU125582.1	18	*Lb. paracasei* strain MG5209	MN720606.1
9	*Lb. brevis* strain ZU 27	AB548882.1	19	*Lb. paracasei* strain NRIC 1940	AB362762.1
10	*Lb. brevis* strain NM101−1	HM218421.1			

### 2.4 Antibacterial activity of LAB isolates

To determine the antibacterial performance of 8 LAB isolates with different RFLP patterns, spot‐on‐the‐lawn method was applied (Koohestani et al., 2018). For this, bacterial suspension was prepared by inoculation of each isolate in MRS broth under anaerobic or aerobic conditions at 37 ± 1°C and then 10 µl of each bacterial suspension was carefully dropped on a plate containing MRS agar. After 48 hr incubation, 7 ml of soft (0.8% w/v agar) trypticase soy broth that previously standardized approximately 6 log_10_ cfu/ml of *L. monocytogenes* ATCC 19,115, by visible‐ultraviolet spectrophotometer at 600 nm, was poured on the palate and inoculated at 37 ± 1°C for 24 hr. Zone of inhibition (ZOI), as a clear area around each spotted bacterial suspension, was checked by a caliper in triplicate. *Lb. casei* 431 was used as a control strain with antibacterial activity.

## 3 RESULTS

### 3.1 Screening and phenotypic characterization of lactic acid bacteria

After incubating each cheese suspension on MRS agar for 24–48 hr at 37°C, various types of bacterial colonies appeared on the surface of MRS agar. A total of 60 MRS agar plates were screened for small, round, matte, and white colonies, of which 70 bacterial colonies showed biochemical properties of LAB including cocci or rods in the shape, Gram‐positive and catalase‐negative.

#### PCR and RFLP

**FIGURE 1:**
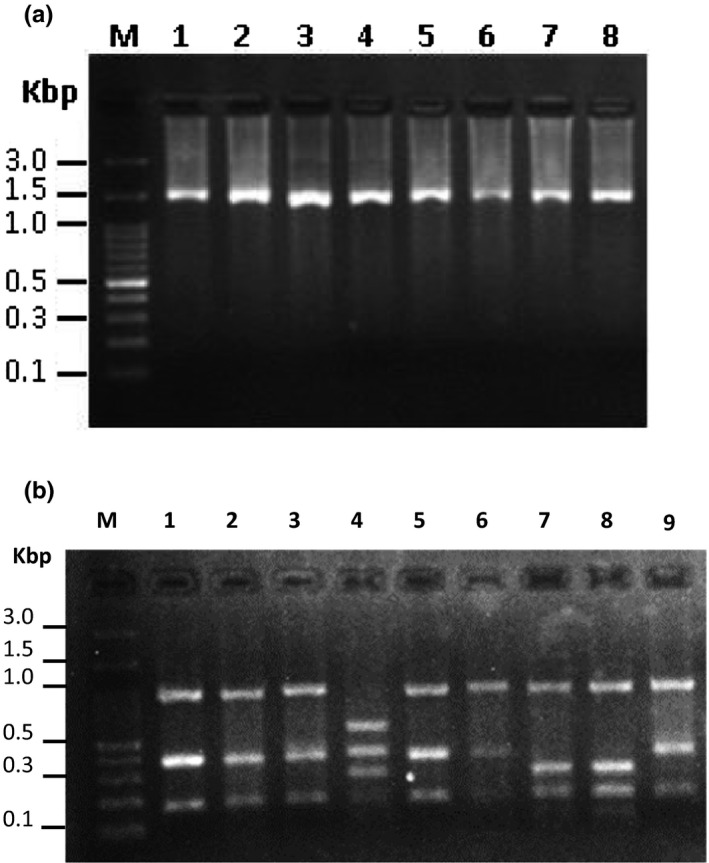
Agarose gel image of amplified fragment of LAB 16s rRNA gene (1,540 bp) using PCR. Lane M, 100 bp molecular ladder; lanes 1–8 amplified PCR products from eight LAB isolates (a); and RFLP patterns of amplified 16S rRNA gene from LAB isolates generated using *Hinf*I endonuclease enzyme. Lane M, 100 bp molecular ladder; lanes 1–3, 5, 6, and 9 RFLP pattern I, lane 4, RFLP pattern 2, and lanes 7 and 8 RFLP pattern 3 (b)

A DNA fragment of 1,540 bp in size was amplified for all isolates from 70 bacterial isolates (Figure 1a). RFLP analysis of the PCR products revealed three different digestion patterns (patterns I‐III) (Figure 1b). Of 70 isolates, amplified fragments of 16s rRNA from 63 (90%) isolates showed RFLP pattern I. RFLP patterns II and III each with two and five isolates were all the RFLP patterns identified using *Hinf*I endonuclease enzyme.

### 3.2 Phylogenetic analysis

**FIGURE 2:**
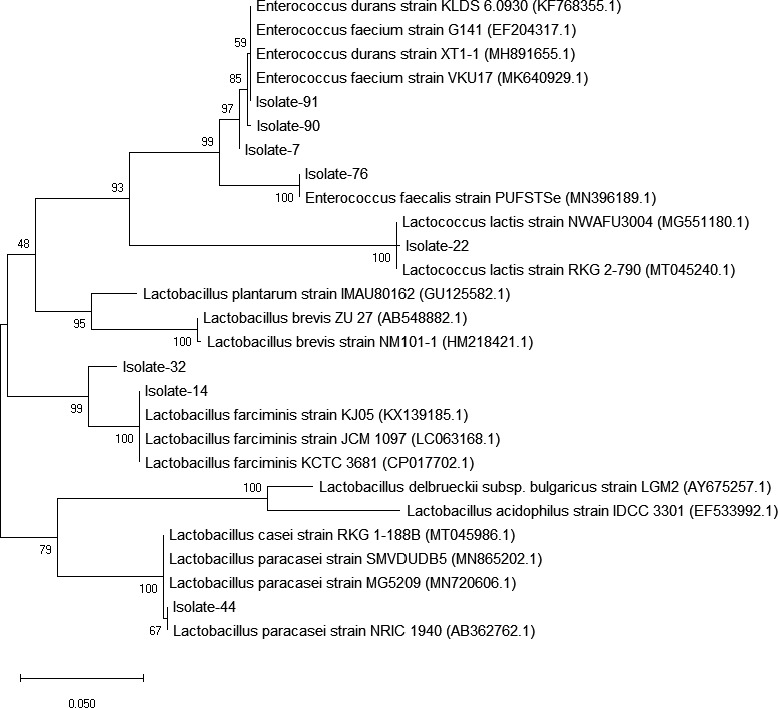
The evolutionary history was inferred using the Neighbor‐Joining method. The optimal tree with the sum of branch length = 0.49584526 was reported. The percentage of replicate trees in which the associated taxa clustered together in the bootstrap test (1,000 replicates) are shown next to the branches. The tree is drawn to scale, with branch lengths in the same units as those of the evolutionary distances used to infer the phylogenetic tree. The evolutionary distances were computed using the p‐distance method and are in the units of the number of base differences per site. This analysis involved 27 nucleotide sequences. Codon positions included were 1st + 2nd+3rd + Noncoding. All ambiguous positions were removed for each sequence pair (pairwise deletion option). There were a total of 616 positions in the final dataset. Evolutionary analyses were conducted in MEGA X (Kumar et al., 2018)

Phylogenetic tree constructed based on neighbor‐joining analysis of 16S rRNA clustered eight examined isolates in four different clusters (Figure 2). Isolates with different RFLP patterns were clustered differently except for isolates 22 and 44 which clustered in two different clusters in the phylogenetic tree. Based on the generated phylogenetic tree, LAB isolates from traditional cheese samples were identified as *Enterococcus* subsp., *Lb. lactis*, *Lb. farciminis*, and *Lb. paracasei*. The most of LAB isolates (90%) from examined cheese samples showing RFLP patter I (isolates 7, 76, 90, 91) belonged to *Enterococcus* subsp. Isolates 14 and 32 showings RFLP pattern II were clustered together. Two isolates 22 and 44 had the same RFLP pattern III; however, based on the phylogenetic tree, these two isolates were clustered in two distinct clusters. *Enterococcus* subsp. were the most prevalent LAB which were identified in the traditional cheeses in the present study.

### 3.3 Antibacterial activity

**FIGURE 3:**
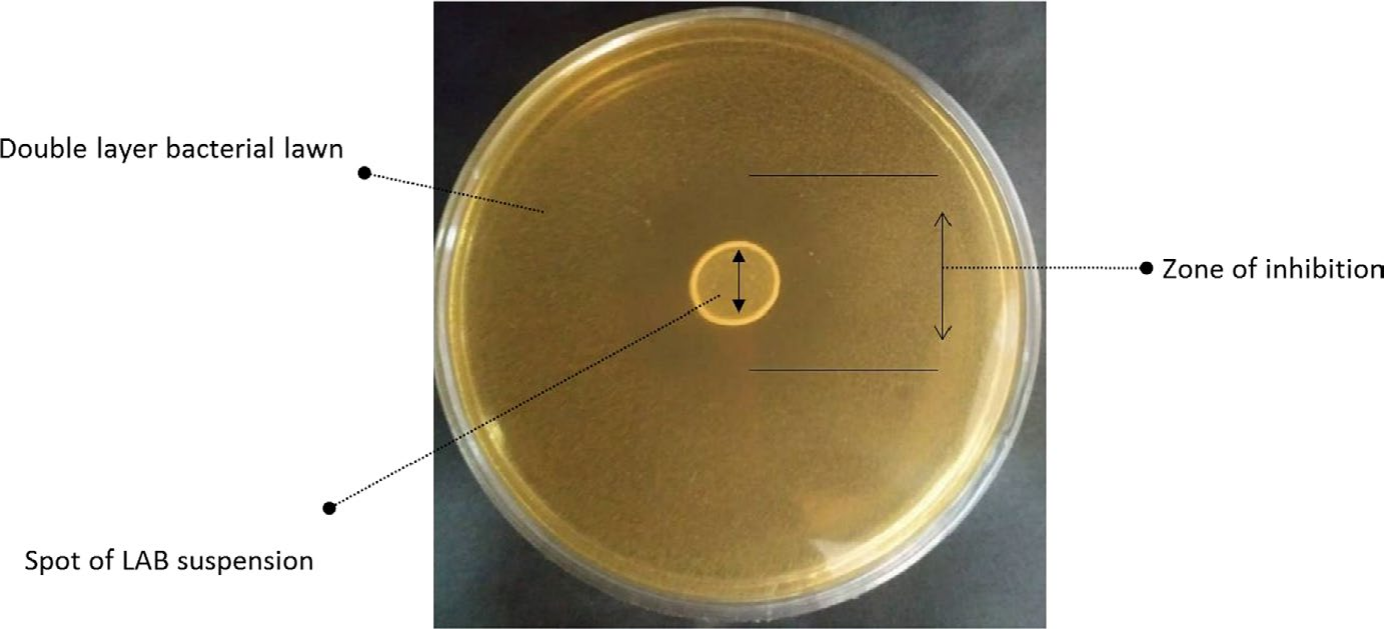
An example of antibacterial zone of inhibition of one selected LAB isolate based on spot‐on‐the‐lawn method

The ZOI (Figure 3) of selected LAB isolates were shown in Table 2. Results showed that antimicrobial performances of all investigated isolates with ZOI were ranged from 6.72 to 14.00 mm, while *Lb. casei* 431 as control strain had 21.00 ± 0.40 mm ZOI. *Lb. paracasei,* which belongs to RFLP pattern III, had the highest ZOI (14.00 mm) among different isolates.

**TABLE 2:** Antibacterial activity of some LAB isolates with different RFLP patterns on *L. monocytogenes* according to spot‐on‐the‐lawn method

Isolate	Species	Identity %	Type of cheese	Incubation condition	RFLP pattern	Antimicrobial activity (ZOI*)
AC7	*Ent. faecium*	98.00	Koopeh	Aerobic	I	9.10 ± 0.30
AC14	*Lb. farciminis*	98.30	Koopeh	Aerobic	II	11.00 ± 0.10
AC22	*Lct. lactis*	99.00	Shoor	Aerobic	III	10.00 ± 0.10
AC32	*Lb. farciminis*	98.00	Shal	Anaerobic	II	11.30 ± 0.10
AC44	*Lb. paracasei*	98.00	Shal	Anaerobic	III	14.00 ± 0.60
AC76	*Ent. faecali*	98.22	Koopeh	Aerobic	I	6.70 ± 0.30
AC90	*Ent. faecium*	93.25	Shoor	Anaerobic	I	9.40 ± 0.20
AC91	*Ent. durans*	98.00	Koopeh	Anaerobic	I	9.20 ± 0.40

* Zone of inhibition.

## 4 DISCUSSIONS

Lactic acid bacteria are a group of beneficial microorganisms that are widely distributed in the nature. They are well known owing to their probiotic potential for food and nutrition application. Probiotic bacteria can confer health benefits to the human gastrointestinal tract. Therefore, nowadays isolation and characterization of LAB strains from different sources is one of the interesting fields of research in the food industry (Alkalbani, Turner, & Ayyash, 2019; Bartkiene et al., 2020). In the present study, the PCR‐RFLP method and phylogenetic analysis based on 16s rRNA gene were used for molecular characterization of LAB isolated from traditional cheese at the species level.

The application of molecular techniques in food microbiology improves our understanding of the ecology and diversity of microbial populations (Hassanzadazar, Mardani, Yousefi, & Ehsani, 2017). Our results confirm the suitability of PCR‐RFLP and phylogenetic analysis for the identification of LAB in food samples at the species level, from which we have obtained useful information on the composition of the microbiota in the traditional cheese. The identification of microbiota of traditional dairy products will help to improve and standardize these products and thus enhance consumer acceptability in broader regions (Yu et al., 2015).

*Enterococcus* subsp. was the most prevalent LAB genus identified in the examined traditional cheese in the present study. The genus *Enterococcus* comprises many species, in which only a few have been studied to be used as probiotics, for example, *Ent. faecalis*, *Ent. faecium*, *Ent. lactis,* and more recently *Ent. hirae* (Adnan, Patel, & Hadi, 2017) and *Ent. durans* (Li et al., 2018). These strains have the ability to produce a variety of antimicrobial compounds, such as organic acids and bacteriocins (Hassanzadazar et al., 2014). In a study by Silva et al. (2015), about 50% of identified LAB isolated from Brazilian water buffalo mozzarella cheese belonged to *Enterococcus* subsp. It was also reported that four strains out of six identified LAB isolates from traditionally fermented Xinjiang cheese were *Ent. hirae* (Azat et al., 2016). Ghahremani, Mardani, and Rezapour (2015) reported that 81% of LAB isolated from traditional cheese in Khorramabad city of Iran belonged to *Enterococcus* subsp. All these studies demonstrated that the majority of LAB isolated from different traditional cheese from various regions have belonged to *Enterococcus* genus. In Russia, seven *Lactobacillus* and *Bifidobacterium* species were identified in the traditional fermented dairy products sampled from different regions (Yu et al., 2015). All these mentioned reports and other works from different countries demonstrating that traditional dairy products have complex compositions of LAB species (Campagnollo et al., 2018; Parsaeimehr, Khazaei, Jebellijavan, & Staji, 2019).

Two strains, *Lb. farciminis* and *Lb. paracasei,* were identified in the examined cheese samples. *Lb. farciminis* is a probiotic strain commercialized by Dupont–Danisco for animal nutrition as a feed additive (Tareb, Bernardeau, Amiel, & Vernoux, 2017). It was shown that *Lb. farciminis* as a feed additive would help to improve ruminal fermentation digestibility without any adverse effect on the pH of rumen and enhance ruminant productivity through manipulation of the rumen microbial ecosystem (Elghandour et al., 2019). *Lb. paracasei* and *Lac. lactis* were the next LAB strains which were identified in the examined cheese in the present study.

Antibacterial potential of LAB is one of the most promising specifications that influence the application of LAB in the food industry (Moradi, Mardani, & Tajik, 2019). Antibacterial agents such as bacteriocins, organic acids, enzymes, alcohols, and low molecular mass substances are among the most studied antimicrobial agents responsible for the antimicrobial action of LAB (Moradi et al., 2020). As reported in this study (Table 2), this activity is species‐ and strain‐dependent. For example, isolates with RFLP pattern of II and III that belong mainly to *Lactococcus* subsp. and *Lactobacillus* subsp. revealed strong antibacterial activity than isolates with RFLP pattern I that consist of *Enterococcus* subsp. Similar results were reported on antibacterial activity of LAB isolated from raw camel milk (Rahmeh et al., 2019), Genestoso cheese (González et al., 2007) and Brazilian artisanal cheeses (Margalho et al., 2020). Organic acids production, as the main antibacterial agents, by LAB is mainly a genus‐specific phenomenon and partially specie‐specific. In this regard, lactic acid production by LAB strains is significantly higher than acetic acid production (Thierry et al., 2015). There is a relationship between organic acid production, type of culture media, and antibacterial activity of LAB. For example, LAB at fish broth and MRS produce succinic and acetic acids at the highest and lowest contents (Özcelik, Kuley, & Özogul, 2016). However, the EPS derived from LAB represent moderate to strong antibacterial activity on some foodborne microorganisms (i.e., pathogens and spoilage groups), depending on bacterial species and EPS source and characterization (Moradi et al., 2020).

## 5 CONCLUSIONS

In conclusion, traditional cheeses examined in the present study were identified as sources of LAB especially *Enterococcus* subsp. It was revealed that PCR‐RFLP combined with 16s rRNA gene sequencing will be a valuable tool for the identification and molecular characterization of LAB in the food commodities especially dairy products. It seems that the observed antibacterial activity appears to be strain‐dependent.

## CONFLICT OF INTEREST

The authors declare no conflict of interest.

## ETHICAL STATEMENTS

This study does not involve any human or animal testing.

## Data Availability

All data are available upon request.
